# Treatment of Punctate Inner Choroidopathy with Choroidal Neovascularization Using Corticosteroid and Intravitreal Ranibizumab

**DOI:** 10.1155/2018/1585803

**Published:** 2018-09-13

**Authors:** Wei Wu, Shiying Li, Haiwei Xu, Yong Liu, Yi Wang, Timothy Y. Y. Lai, Zheng Qin Yin

**Affiliations:** ^1^Southwest Hospital/Southwest Eye Hospital, Third Military Medical University (Army Medical University), Chongqing, China; ^2^Department of Ophthalmology & Visual Sciences, Chinese University of Hong Kong, Hong Kong

## Abstract

**Background:**

To evaluate the treatment outcomes of patients with punctate inner choroidopathy (PIC) and secondary choroidal neovascularization (CNV).

**Methods:**

This is a retrospective study of 24 eyes in 22 patients suffering from PIC with CNV. Patients were treated with intravitreal ranibizumab monotherapy (14 eyes) or combined oral corticosteroid and intravitreal ranibizumab therapy (corticosteroid-ranibizumab group, 10 eyes). Mean follow-up duration was 24.0 months. We evaluated best-corrected visual acuity (BCVA), fundus autofluorescence, fluorescein angiography, indocyanine green angiography, and optical coherence tomography, before and after treatment. The following variables were compared between groups: number of intravitreal ranibizumab injections, BCVA, recurrence of CNV, and change in PIC lesions.

**Results:**

The ranibizumab monotherapy group received an average of 3 intravitreal ranibizumab injections; mean logMAR visual acuity improvement was 0.34, and 8 eyes developed recurrent CNV during follow-up. The corticosteroid-ranibizumab group received an average of 1.9 intravitreal ranibizumab injections; mean logMAR visual acuity improvement was 0.61, and there was no recurrence of CNV. Combined corticosteroid-ranibizumab therapy also resulted in better resolution of PIC lesions and fewer new PIC lesions.

**Conclusion:**

Both corticosteroid-ranibizumab treatment and ranibizumab monotherapy could significantly improve the vision of PIC patients with CNV. Combined corticosteroid and intravitreal ranibizumab treatment appeared to reduce CNV recurrence and development of new PIC lesions compared with ranibizumab monotherapy.

## 1. Background

Punctate inner choroidopathy (PIC) is a specific form of posterior uveitis, firstly described by Watzke and colleagues in 1984 [1]. The disease most commonly affects young, myopic women and is characterized by multifocal, well-circumscribed, yellow-white choroidal lesions at the posterior pole of the retina, without anterior or vitreous inflammation. Subfoveal lesions can result in symptoms including blurred vision, scotoma, metamorphopsia, and photopsia [2, 3]. PIC does not usually cause severe visual loss until development of choroidal neovascularization (CNV), but CNV can develop in up to 70% of patients with PIC [2, 4].

Considering that PIC is a kind of ocular inflammatory disorder, corticosteroid is recommended to reduce inflammation. For PIC patients without CNV, oral corticosteroid may help to achieve more rapid visual improvement although spontaneous resolution of the lesions can occur in many cases without treatment [5]. However, there is no consensus on the optimal treatment for PIC with CNV. A number of treatment modalities have been described, including thermal focal laser photocoagulation, surgical CNV extraction, and verteporfin photodynamic therapy (vPDT). However, these have demonstrated limited visual benefit, due to high rates of CNV recurrence, or irreversible damage to the retinal pigment epithelium (RPE) [6, 7].

More recently, antivascular endothelial growth factor (anti-VEGF) agents, such as ranibizumab, bevacizumab, and conbercept, have been used to treat CNV secondary to PIC, and they have appeared to be safe and effective [8–13]. We therefore hypothesized that the combination of oral corticosteroid and intravitreal ranibizumab could both reduce inflammation in PIC and treat secondary CNV. Here, we retrospectively studied 24 eyes, in 22 patients suffering from PIC with CNV, and compared combined oral corticosteroid-ranibizumab therapy with intravitreal ranibizumab monotherapy.

## 2. Methods

### 2.1. Study Design

We retrospectively studied the clinical records of 22 patients suffering from PIC with secondary CNV who attended the Southwest Eye Hospital, Chongqing, China, from June 2009 to September 2017. This study was performed in accordance with the tenets of the Declaration of Helsinki and was approved by the ethics committee of Southwest Hospital, Third Military Medical University. Consent was obtained from all patients for use of their clinical data for this study.

A diagnosis of PIC was made by two experienced retinal specialists, independently, on the basis of clinical examination, fluorescein angiography (FA) and/or indocyanine green angiography (ICGA), fundus autofluorescence (FAF), and optical coherence tomography (OCT). PIC lesions were classified into five stages, according to the method by Zhang and colleagues [2, 14]. In order to exclude other causes of posterior uveitis, all patients underwent laboratory workup and imaging studies including chest X-ray, detection of systemic infections such as toxoplasmosis, syphilis, varicella-zoster, and herpes, and antibody against mycobacterium tuberculosis. Other exclusion criteria for the current study included patients with follow-up of less than three months and patients with CNV secondary to other causes (such as age-related macular degeneration, polypoidal choroidal vasculopathy, pathologic myopia, and angioid streaks).

Following diagnosis of PIC with CNV, all patients were advised to receive combination therapy with oral corticosteroid and intravitreal ranibizumab, and 8 patients (2 with bilateral disease) proceeded with this regime. Fourteen patients refused corticosteroid due to potential side effects and were therefore only given ranibizumab monotherapy. This provided us with a combination corticosteroid-ranibizumab treatment group (n = 10 eyes) and a ranibizumab monotherapy group (n = 14 eyes), for retrospective comparison of outcome.

Intravitreal ranibizumab monotherapy was performed using a single intravitreal injection of 0.5 mg ranibizumab (Lucentis, Novartis, Basel, Switzerland) in 0.05 mL water for injections, followed by as needed injections, as judged necessary by the responsible ophthalmologist. The corticosteroid-ranibizumab group additionally received oral prednisolone (1 mg/kg per day initially, then weaned by 10 mg every 14 days). To minimize the potential side effects of corticosteroids, patients were treated with oral prednisone for no more than 3 months.

Follow-up OCT and FAF were performed after treatment to assess the activity and progression of PIC and to determine further treatment. When necessary, additional FA and/or ICGA were performed to determine the activity of PIC and CNV during follow-up. Retreatment with ranibizumab was administered in cases with persistent or recurrent intraretinal edema, or subretinal fluid demonstrated by OCT, and/or hemorrhage from CNV demonstrated by FA.

### 2.2. Statistical Analysis

For statistical analysis, Snellen best-corrected visual acuity (BCVA) was converted to the logarithm of the minimum angle of resolution (logMAR) equivalents. We used the unpaired (independent) two-sample* t*-test to compare the means between the two groups and the Mann–Whitney* U* test where parametric test assumptions were not satisfied. For within-group comparisons (e.g., between two time-points) we used the paired* t*-test. A P value of < 0.05 was considered to be statistically significant.

## 3. Results

### 3.1. Patient Demographics

Twenty-four eyes of 22 patients (6 male and 16 female) were included in the study. Twenty patients (90.9%) had unilateral disease and 2 (9.1%) cases (both of whom were in the corticosteroid-ranibizumab group) had bilateral PIC. The mean ± standard deviation (SD) age of the patients at presentation was 38.3 ± 11.7 years (range, 22–65 years). Notably, the mean ± SD age of the patients in ranibizumab monotherapy group (42.9 ± 11.4) was significantly higher than that in the corticosteroid-ranibizumab group (30.0 ± 8.0) (P = 0.006, unpaired* t*-test). Of the 24 eyes, 23 (95.8%) eyes had myopia of −2.25 D or more. Fourteen eyes received ranibizumab monotherapy and 10 eyes received combined corticosteroid and ranibizumab therapy (Table 1). The mean (± SD) number of ranibizumab injections given in the corticosteroid-ranibizumab group (1.9 ± 1.0 injections; range, 1–3) was less than that in the ranibizumab monotherapy group (3 ± 1.7 injections; range, 1–7), although this difference was not significant (P = 0.102, Mann–Whitney* U* test). Mean follow-up duration was 24 months (range, 3–52 months) across all patients, and there was no difference between the ranibizumab monotherapy group (23.4 ± 16.0 months) and the corticosteroid-ranibizumab group (24.5 ± 10.0) (P = 0.844, unpaired* t*-test).

### 3.2. Visual Acuity and Recurrence of CNV

**Figure 1:**
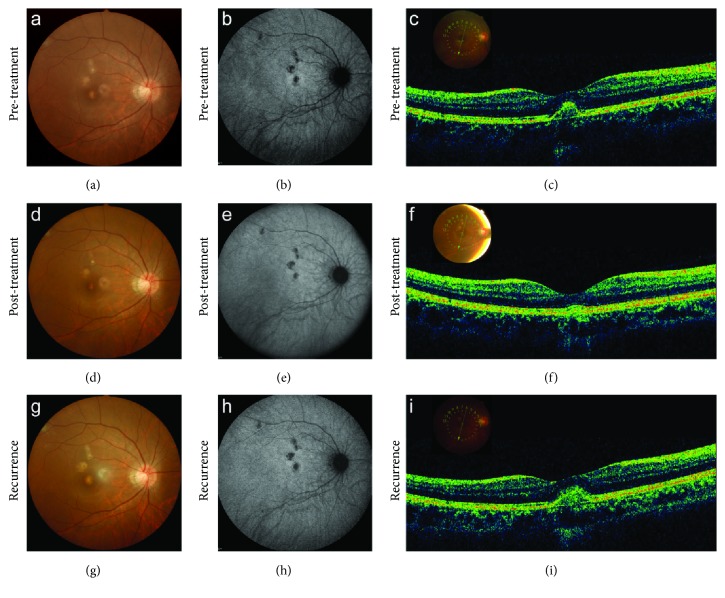
(a) Fundus photograph of the right eye of a 36-year-old female PIC patient at the initial presentation showing 5 yellow-white lesions of PIC and subfoveal hemorrhage. The logMAR BCVA at presentation was 0.52. (b) FAF showed well-defined hypofluorescence in the lesions. (c) OCT showed the presence of CNV and intraretinal fluid. (d-f) Fundus photograph, FAF, and OCT examination 3 months after 3 ranibizumab monthly intravitreal injections. The patient's CNV resolved and logMAR BCVA increased to 0.1. (g-i) Fundus photograph, FAF, and OCT examination 8 months later. Recurrent CNV was noted and logMAR BCVA decreased to 0.4.

Table 1 shows the treatment outcomes of all 24 eyes. In the ranibizumab monotherapy group (14 eyes), the mean (± SD) logMAR BCVA at baseline was 0.68 ± 0.46. At the last follow-up, the mean logMAR BCVA improved significantly to 0.33 ± 0.39 (P = 0.02, paired* t*-test). In the corticosteroid-ranibizumab therapy group (10 eyes), the mean (± SD) logMAR BCVA improved significantly from 0.82 ± 0.52 at baseline to 0.22 ± 0.23 at the last visit (P = 0.01, paired* t*-test). Comparing the two groups, the initial mean BCVA was not significantly different (P = 0.378, Mann–Whitney* U* test). In addition, the mean improvement (± SD) in logMAR BCVA in the corticosteroid-ranibizumab group (0.61 ± 0.43) was higher than that seen in the ranibizumab monotherapy group (0.34 ± 0.46), although the difference between the two groups was not significant (P = 0.174, unpaired* t*-test). However, three months after the first ranibizumab treatment, 8 eyes (57.1%) developed recurrence of CNV in the ranibizumab monotherapy group (e.g., Figure 1), whereas no eyes developed recurrence in the corticosteroid-ranibizumab group.

### 3.3. Anatomical Outcome of PIC Lesions

**Figure 2:**
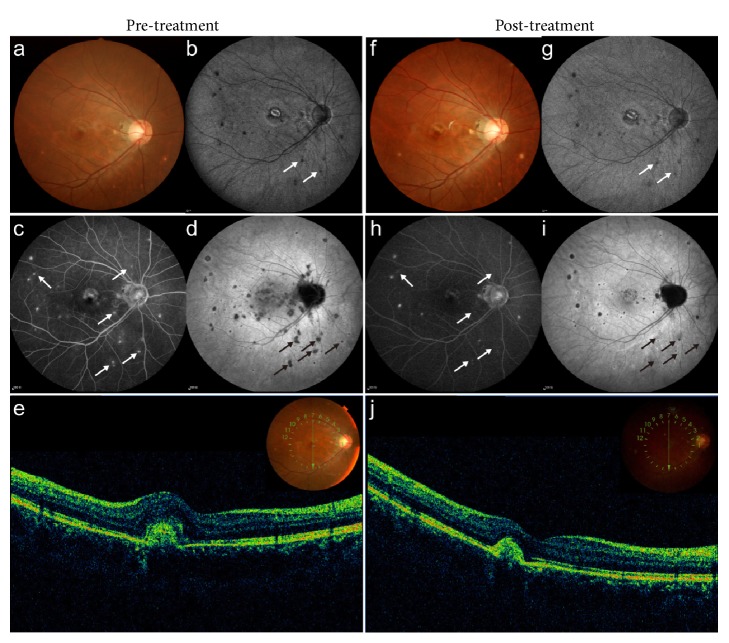
(a) Fundus photograph of the right eye of a 28-year-old female patient with PIC and CNV at initial presentation, showing yellow-white choroidal lesions and subfoveal hemorrhage. The logMAR BCVA was 0.7. (b) FAF image showed clear hypofluorescence of the lesions. (c) Late-phase FA image detected hyperfluorescent lesions. (d) Late-phase ICGA image detected areolar hyperfluorescence surrounding central dark spots. (e) OCT image demonstrated the presence of CNV and intraretinal fluid. (f) Fundus photograph at 12 months after initiating combined oral corticosteroid and ranibizumab therapy. The logMAR BCVA increased to 0. (g) FAF image showed less hypofluorescent lesions posttreatment. Note an evident resolution of the lesions compared with the first visit (white arrows). (h) Late-phase FA image showed less hyperfluorescent lesions after treatment (white arrows). (i) Late-phase ICGA image showed dramatic resolution and shrinkage of hypofluorescent lesions posttreatment (black arrows). (j) OCT showed the resolution of the CNV and intraretinal fluid.

We next used the number of PIC lesions as an outcome measure. PIC lesions in the 24 eyes included in this study were all classified as being stage III or IV. Specifically, fundal examination showed yellow-white creamy spots (e.g., Figure 2(a)). An even larger number of hypofluorescent spots were typically evident with FAF (e.g., Figure 2(b)). FA typically showed multifocal hyperfluorescent spots (e.g., Figure 2(c)), which appeared at ICGA as areolar hyperfluorescence around central dark spot (e.g., Figure 2(d)). OCT typically revealed subfoveal CNV, with reflectivity defects throughout the RPE (e.g., Figure 2(e)). After combined corticosteroid-ranibizumab therapy, both CNV and PIC lesions typically improved (e.g., Figures 2(f)–2(j)). Furthermore, in 2 eyes (20%), the number of PIC lesions was reduced; in 6 eyes (60%), the number was stable; and only 2 (20%) eyes developed new lesions. By comparison, after ranibizumab monotherapy, none of the eyes (0%) had reduced numbers of lesions, 8 eyes (57.2%) had a stable number of lesions, and 6 eyes (42.8%) developed new lesions.

### 3.4. Adverse Events

Following treatment, we did not observe any instances of endophthalmitis, retinal detachment, glaucoma, any other serious ocular adverse events, or systemic adverse events.

## 4. Discussion

Patients with PIC generally have good visual prognosis unless the lesions are subfoveal or secondary CNV develops [1, 4, 16]. PIC can be considered as one of the clinical phenotypes seen among patients with multifocal choroiditis, and immunosuppressive therapy might therefore have a role in the treatment of PIC [17, 18]. Subfoveal CNV can result in significant visual loss, due to irreversible damage to the neurosensory retina and RPE. Treatment options for CNV secondary to PIC include thermal laser photocoagulation, submacular surgery, oral corticosteroid (or other immunomodulators), vPDT, and intravitreal anti-VEGF therapy [19]. Among these treatment options, vPDT and intravitreal anti-VEGF injection are currently the most widely used.  Table 2 summarizes several studies on the treatment of CNV secondary to PIC. Whilst vPDT treatment has been shown to be beneficial for stabilizing and also improving vision in some patients with PIC and CNV, one-third of eyes have persistent poor visual acuity [15]. vPDT may also cause collateral damage to the retinal and RPE layers, so visual outcomes can be inconsistent [7].

VEGF has a pivotal role in the development of CNV, and anti-VEGF therapy has been shown to be safe and effective as a first-line treatment for CNV due to ocular inflammatory like PIC [11–13]. In a previous retrospective study of 10 eyes suffering from PIC with CNV, the authors report that intravitreal ranibizumab monotherapy improved or stabilized vision in 9 eyes, with a mean follow-up period of 12.5 months [11]. Here we report findings from 14 eyes receiving ranibizumab monotherapy, with a mean follow-up of 23.3 months, and have shown that, whilst anti-VEGF monotherapy did improve vision, 8 eyes (57.1%) developed recurrent CNV.

Because inflammation contributes to the development of PIC and secondary CNV, anti-inflammatory treatments play a key role in reducing or eradicating the stimulus that leads to CNV formation [6]. It is thought that corticosteroid can reduce development of CNV by inhibiting both VEGF [20, 21] and matrix metalloproteinases [22], thus providing both anti-inflammatory and antiangiogenic properties. However, there is still debate about whether PIC associated with CNV should be treated with corticosteroid [23]. In our study, we therefore studied the benefit of combining oral prednisolone and intravitreal ranibizumab to treat PIC with CNV. None of the 10 eyes treated using this regime developed recurrent CNV. Patients receiving combined corticosteroid−anti-VEGF appeared to have greater improvement in vision and to require fewer intravitreal anti-VEGF injections compared with anti-VEGF monotherapy. However, these results were not statistically significant, and the retrospective, nonrandomized nature of this study means we cannot exclude the effect of biases and confounders. Consistent with our findings, previous small-scale case series (less than 3 patients) have reported that combining corticosteroid and anti-VEGF therapy should be considered as a management of CNV associated with PIC [24, 25]. In addition to corticosteroids, immunosuppressants have also been proposed for control of inflammatory CNV [26]. Considering the side effects, administration of corticosteroids and immunosuppressants should be based on the patients' general and ocular conditions. Notably, combination of corticosteroids with immunosuppressive drugs (e.g., mycophenolate mofetil) is recommended for the CNV unresponsive to traditional immunosuppressants [27].

In our study, we also evaluated the potential therapeutic effects of corticosteroid on PIC itself. To thoroughly analyze the changes in PIC lesions, a dual FA/ICGA imaging is strongly encouraged [28]. As shown in Figure 2, both FA and ICGA image showed a dramatic resolution and shrinkage of PIC lesions after combined corticosteroid-ranibizumab therapy. Compared with the 14 eyes in the anti-VEGF monotherapy group, the 10 eyes which received combined therapy had better resolution of PIC lesions and developed fewer new lesions. This is consistent with a previous study, which has shown that oral corticosteroid promotes the resolution of PIC lesions [5].

## 5. Conclusions

Our results demonstrate that both corticosteroid-ranibizumab treatment and ranibizumab monotherapy can significantly improve the vision of PIC patients with CNV. Combined corticosteroid and intravitreal ranibizumab treatment appeared to reduce CNV recurrence and development of new PIC lesions compared with ranibizumab monotherapy. Clearly the strength of these conclusions is limited by the retrospective nature of this study and the small sample size. Ideally, a prospective, randomized placebo-controlled trial would be performed, with a longer follow-up duration, to more conclusively determine the role of combined oral corticosteroid and anti-VEGF as first-line treatment for PIC with secondary CNV.

## Figures and Tables

**Table 1:** Patient characteristics and responses to treatment.

	**Intravitreal ranibizumab monotherapy**	**Combined corticosteroid and ranibizumab**	**P value**
	**(n = 14 eyes)**	**(n = 10 eyes)**	
Mean age	42.9	30.0	0.006
Mean follow-up	23.3 months	24.5 months	0.844
Initial BCVA	0.68 logMAR	0.82 logMAR	0.378
Final BCVA	0.34 logMAR	0.22 logMAR	
Change of BCVA	-0.34 logMAR	-0.61 logMAR	0.174
Mean intravitreal injections given	3.0	1.9	0.102
Recurrent CNV (eyes)	8 (57.1%)	0 (0%)	
Improved or stable BCVA (eyes)	13 (92.9%)	10 (100%)	
Reduced lesions (eyes)	0 (0%)	2 (20%)	
Stable lesions (eyes)	8 (57.2%)	6 (60%)	
New lesions (eyes)	6 (42.8%)	2 (20%)	

Improved or stable BCVA means that BCVA of the patient kept stable (less than 0.1 in change) or was improved to more than 0.1 after the treatment.

**Table 2:** Comparison of studies on the treatment of CNV secondary to PIC.

	**Coco et al. [15]**	**Peng et al. [12]**	**Fong et al. [6]**	**Menezo et al. [11]**	**Zhang et al. [8]**	**Our study**
Study design	Retrospective	Prospective	Prospective	Retrospective	Prospective	Retrospective
No. of eyes	8	16	5	10	12	24
Treatment	vPDT	Conbercept	vPDT+ Corticosteroid	Ranibizumab	Bevacizumab	Ranibizumab +/- Corticosteroid
Initial BCVA (logMAR)	0.42	0.70	0.54	0.72	0.49	Ranibizumab: 0.68; Ranibizumab+ Corticosteroid: 0.82
Final BCVA (logMAR)	0.51	0.44	0.39	0.37	0.23	Ranibizumab: 0.34; Ranibizumab+ Corticosteroid: 0.22
Mean follow-up (months)	22.7	6	12	12.5	12	Ranibizumab: 23.3; Ranibizumab+ Corticosteroid: 24.5
Improved or stable BCVA (eyes)	5 (62.5%)	16 (100%)	4 (80%)	9 (90%)	12 (100%)	Ranibizumab: 13 (92.9%); Ranibizumab+ Corticosteroid: 10 (100%)

Improved or stable BCVA means that BCVA of the patient kept stable (less than 0.1 in change) or was improved to more than 0.1 after the treatment.

## Data Availability

The datasets used and/or analyzed during the current study are available from the corresponding author on reasonable request.

## Abbreviations

PIC: Punctate inner choroidopathy
CNV: Choroidal neovascularization
vPDT: Verteporfin photodynamic therapy
RPE: Retinal pigment epithelium
VEGF: Vascular endothelial growth factor
FA: Fluorescein angiography
ICGA: Indocyanine green angiography
FAF: Fundus autofluorescence
OCT: Optical coherence tomography
BCVA: Best-corrected visual acuity
LogMAR: Logarithm of the minimum angle of resolution
SD: Standard deviation.
